# Application of genetic risk score for in-stent restenosis of second- and third-generation drug-eluting stents in geriatric patients

**DOI:** 10.1186/s12877-023-04103-w

**Published:** 2023-07-19

**Authors:** Yu-Ling Hsu, Mu-Shiang Huang, Hsien-Yuan Chang, Cheng-Han Lee, Dao-Peng Chen, Yi-Heng Li, Ting-Hsin Chao, Yen-Wen Liu, Ping-Yen Liu

**Affiliations:** 1 Division of Cardiology, Department of Internal Medicine, National Cheng Kung University Hospital, College of Medicine, National Cheng Kung University, Tainan, 704 Taiwan; 2 Institute of Clinical Medicine, College of Medicine, National Cheng Kung University, 138 Sheng-Li Rd. North District, Tainan, 704 Taiwan; 3 KimForest Enterprise Co., Ltd, New Taipei City, 221 Taiwan

**Keywords:** Geriatric patients, Drug-eluting stent, In-stent restenosis, Genetic risk score, Single nucleotide polymorphism

## Abstract

**Background:**

The second-and third-generation drug-eluting stents (DESs) in-stent restenosis (ISR) genetic risk score (GRS) model has been previously validated. However, the model has not been validated in geriatric patients. Therefore, we conducted this study to test the feasibility of the DES-ISR GRS model in geriatric patients with coronary artery disease (CAD) in Taiwan.

**Methods:**

We conducted a retrospective, single-center cohort study and included geriatric patients (age ≥ 65 years) with CAD and second-or third-generation DES(s) deployment. Patients undergoing maintenance dialysis were excluded. ISR was defined as ≥ 50% luminal narrowing on the follow-up coronary arteriography. The DES-ISR GRS model included five selected exonic single-nucleotide polymorphisms (SNPs): CAMLG, GALNT2, C11orf84, THOC5, and SAMD11. The GRS was defined as the sum of the five selected SNPs for the risk allele.

**Results:**

We enrolled 298 geriatric patients from January 2010 and December 2019 in this study. After propensity score matching, there were 192 geriatric patients with CAD in the final analysis, of which 32 patients had ISR. Patients were divided into two groups based on their GRS values: low (0–2) and high (≥ 3) GRS. A high GRS was significantly associated with DES-ISR in geriatric patients.

**Conclusion:**

Those geriatric patients with a high GRS had significantly higher second-or third-generation DES ISR rates. The five SNP-derived DES-ISR GRS model could provide genetic information for interventional cardiologists to treat geriatric patients with CAD.

**Trial registration:**

The primary study protocol was registered with clinicaltrials.org. with registration number: NCT03877614; on March 15, 2019. (http://clinicaltrials.gov/ct2/show/NCT03877614)

## Introduction

Coronary artery stents provide mechanical scaffolding to decrease the impact of negative coronary artery remodeling. Post-stenting neointimal hyperplasia emerges as an unfavorable result leading to in-stent restenosis (ISR). The material amelioration of the second-and third-generation drug-eluting stents (DESs) significantly suppresses neointima hyperplasia and reduces the incidence of DES-ISR compared to bare-metal stents (BMSs) and first-generation DESs. Nevertheless, the second-and third-generation DES-ISR could not be completely eliminated and remains a challenge [1]. Although several factors, including aging, mechanical, and biological, can be attributed to second-and third-generation DES-ISR [1, 2], the genetic factors associated with DES-ISR have not been elucidated. We identified five exonic single-nucleotide polymorphisms (SNPs) significantly associated with second- and third-generation DES-ISR and constructed a five-SNP-derived DES-ISR genetic risk score (GRS) model for testing in East Asians [3]. With age, there would be more comorbidities: such as hypertension, hyperlipidemia, and chronic kidney disease, which might contribute to neo-atherosclerosis. Furthermore, some of the mechanisms (e.g., inflammation) resulting in vascular aging also participate in the pathophysiologic process of neointima hyperplasia. Due to these complex and unique natures, the genetic impact on the DES-ISR in the geriatric population is uncertain and has been seldom reported. The application of this DES-ISR GRS model in the geriatric population has not been investigated till date. Therefore, we conducted this study to evaluate the power of the DES-ISR GRS model to detect second-and third-generation DES-ISR in older people.

## Methods

### Study design

This was a retrospective, single-center study. The study design, demographic characteristics, comorbidities, medication history, percutaneous coronary intervention information, and exonic SNP analysis methods and results have been published previously [3]. Additionally, DES-ISR was defined as follows: ≥ 50% stent luminal narrowing or within 5 mm of a stent edge at follow-up coronary arteriography [3, 5]. Three qualified interventionists reviewed the medical records and coronary angiographic images to confirm DES-ISR events.

Using the established five-SNP-derived DES-ISR GRS model (consisting of SNPs in *CAMLG*, *GALNT2*, *C11orf84*, *THOC5*, and S*AMD11*) [3], we calculated the GRS of every older patient in this study. The number of risk alleles for each exonic SNP, ranging from 0 to 2, was assigned to each SNP to calculate the DES-ISR GRS. Thus, the DES-ISR GRS would be equal to the sum of the risk allele numbers of these five SNPs [3].

### Study population

A coronary artery disease cohort included 2,749 patients with new-generation DES deployment who were admitted at a Taiwanese university medical center from January 2010 to December 2019. After the screening, 690 patients would like to participate in genomic research, and 92 patients had DES ISR. We excluded 60 patients, including 54 dialysis patients and 6 patients with missing clinical information. Finally, 630 patients were recruited for analysis to build the GRS model [3]. We conducted a subgroup analysis to demonstrate the feasibility of the GRS model for DES-ISR risk prediction in older people (age ≥ 65 years). This study was conducted according to the principles of the Declaration of Helsinki and approved by the NCKUH Human Research and Ethics Committee (IRB: A-ER-107-049). Written informed consent was obtained from all enrolled patients. The study protocol was also registered with clinicaltrials.org (http://clinicaltrials.gov/ct2/show/NCT03877614) [3, 4].

### Definition of second-and third-generation DESs

The second-and third-generation DESs were defined as everolimus-eluting stents with a durable polymer (Xience [Abbott Vascular, Santa Clara, California] and Promus [Boston Scientific, Natick, Massachusetts]), zotarolimus-eluting stents with a durable polymer (Resolute [Medtronic, Minneapolis, Minnesota]), everolimus-eluting stents with a bioabsorbable polymer (Synergy [Boston Scientific, Natick, Massachusetts]), biolimus-eluting stents with a biodegradable polymer (Nobori [Terumo, Tokyo, Japan]), Ultimaster sirolimus-eluting stents with a biodegradable polymer (Ultimaster [Terumo, Tokyo, Japan] and Orsiro [Biotronik, Bülach, Switzerland]), Biomatrix biolimus A9 (BA9)-eluting stents with a biodegradable polymer, and BioFreedom BA9-coated stents (Biosensors, Newport Beach, California) [3].

### Statistical analysis

Continuous and dichotomous data are expressed as median (IQR) and numbers (percentages), respectively. Student’s *t*-tests were applied to compare normally distributed continuous variables, and nonparametric tests were used to compare continuous variables that were not normally distributed. Fisher’s exact test was used to analyze categorical variables. Baseline confounding factors between the DES-ISR (+) and DES-ISR (-) groups were adjusted by applying propensity score matching. A diagnostic accuracy test was performed to determine the cut-off value of the DES-ISR GRS. Univariate Cox regression analysis was performed to evaluate factors associated with DES-ISR. Those factors with *p* < 0.1 based on univariate Cox regression analysis were included in the multivariate Cox regression analysis to identify independent factors of the DES-ISR. The Kaplan–Meier method with log-rank tests was used to compare the DES-ISR-free rates between the two groups. Furthermore, Cox regression analysis was applied to determine the hazard ratio (HR) of the GRS and the risk of ISR. Statistical significance was set at *p* < 0.05.

## Results

A total of 298 geriatric patients with CAD who received second-and third-generation DESs deployment from January 2010 to December 2019 were included. Of the 298 patients, 32 had DES-ISR, which was confirmed by coronary arteriography. However, there was a significant difference in the old myocardial infarction history between the DES-ISR (-) and DES-ISR (+) groups (*p* = 0.002, Table 1). Therefore, we performed propensity score matching to eliminate this difference. After propensity score matching, 192 geriatric patients (32 with DES-ISR) were enrolled in the geriatric cohort. There were no significant differences in the baseline demographic characteristics (Table 1).

**Table 1:** Baseline clinical demographics of the geriatric cohort with or without drug-eluting stent (DES) instent restenosis (ISR)

	No propensity score matching				After propensity score matching		
	DES ISR (-) (n = 266)	DES ISR (+) (n = 32)	*p*		DES ISR (-) (n = 160)	DES ISR (+) (n = 32)	*p*
Age (years)	77.82 (73.8–82.5)	78.21 (74.43–83.24)	0.97		77.99 (74.13–82.61)	78.21 (74.43–83.24)	0.88
Male, n (%)	195 (73.3%)	24 (75%)	> 0.99		117 (73.1%)	20 (83.3%)	> 0.99
Comorbidities							
Diabetes mellitus, n (%)	125 (47%)	16 (50%)	0.85		74 (46.2%)	10 (41.7%)	0.70
Dyslipidemia, n (%)	200 (75.2%)	26 (81.2%)	0.52		128 (80%)	20 (83.3%)	> 0.99
Hypertension, n (%)	179 (67.3%)	23 (71.9%)	0.69		107 (66.9%)	14 (58.3%)	0.68
Old stroke, n (%)	20 (7.5%)	2 (6.2%)	> 0.99		12 (7.5%)	0 (0%)	> 0.99
CAD, n (%)	261 (98.1%)	31 (96.9%)	0.50		156 (97.5%)	22 (91.7%)	> 0.99
CKD, n (%)	54 (20.3%)	10 (31.2%)	0.17		44 (27.5%)	6 (25%)	0.67
Atrial fibrillation, n (%)	45 (16.9%)	8 (25%)	0.33		34 (21.2%)	3 (12.5%)	0.64
Old MI, n (%)	76 (28.6%)	18 (56.2%)	0.002		76 (47.5%)	14 (58.3%)	0.44
Heart failure, n (%)	52 (19.5%)	8 (25%)	0.49		38 (23.8%)	8 (25%)	0.83
PAOD, n (%)	2 (0.8%)	0 (0%)	> 0.99		2 (1.2%)	1 (4.2%)	0.60
Medication							
Aspirin, n (%)	218 (82%)	27 (84.4%)	> 0.99		135 (84.4%)	20 (83.3%)	> 0.99
Clopidogrel, n (%)	205 (77.1%)	23 (71.9%)	0.51		119 (74.4%)	17 (70.8%)	0.83
Ticagrelor, n (%)	27 (10.2%)	5 (15.6%)	0.36		25 (15.6%)	7 (29.2%)	> 0.99
Apixaban, n (%)	1 (0.4%)	0 (0%)	> 0.99		1 (0.6%)	0 (0%)	> 0.99
Edoxaban, n (%)	1 (0.4%)	0 (0%)	> 0.99		1 (0.6%)	0 (0%)	> 0.99
Rivaroxaban, n (%)	13 (4.9%)	1 (3.1%)	> 0.99		9 (5.6%)	1 (3.1%)	> 0.99
Dabigatran, n (%)	2 (0.8%)	0 (0%)	> 0.99		2 (1.3%)	2 (8.3%)	> 0.99
Warfarin, n(%)	7 (2.6%)	0 (0%)	> 0.99		2 (1.3%)	0 (0%)	> 0.99
ACEi / ARB, n(%)	116 (43.6%)	15 (46.9%)	0.85		78 (48.8%)	13 (54.2%)	> 0.99
β-blocker, n (%)	85 (32%)	12 (37.5%)	0.55		51 (31.9%)	12 (37.5%)	0.54
DHP-CCB, n (%)	40 (15%)	9 (28.1%)	0.08		21 (13.1%)	9 (28.1%)	0.06
Statin, n (%)	174 (65.4%)	17 (53.1%)	0.18		110 (68.8%)	16 (66.7%)	0.10

Exonic SNPs analysis was performed in our CAD cohort as previously published [3]. We identified five high-impact SNPs: *CAMLG*, *GALNT2*, *C11orf84*, *THOC5*, and S*AMD11*. In this geriatric subgroup analysis, the five selected SNPs were significantly associated with DES-ISR (Table 2).

**Table 2:** Details of eligible exonic SNP of 2nd - or 3rd -generation DES-ISR GRS model in geriatric coronary artery disease patients after propensity score matching

Gene	RSID	Risk allele	Non-risk allele	Risk allele frequency	DES ISR (-)		DES ISR (+)		ISR Odds ratio
					Risk allele, n	Non-risk allele, n	Risk allele, n	Non-risk allele, n	
CAMLG	rs12657663	T	C	0.22	50	108	19	13	3.16
GALNT2	rs2273970	A	G	0.34	94	66	27	5	3.79
C11orf84	rs643634	C	A	0.32	84	75	18	14	1.15
THOC5	rs737976	C	T	0.23	58	102	20	12	2.93
SAMD11	rs9988179	A	G	0.11	28	132	6	26	1.09

Abbreviation: DES: drug−eluting stent; GRS: genetic risk score; ISR: in−stent restenosis; SNP: single nucleotide polymorphism

**Fig. 1:**
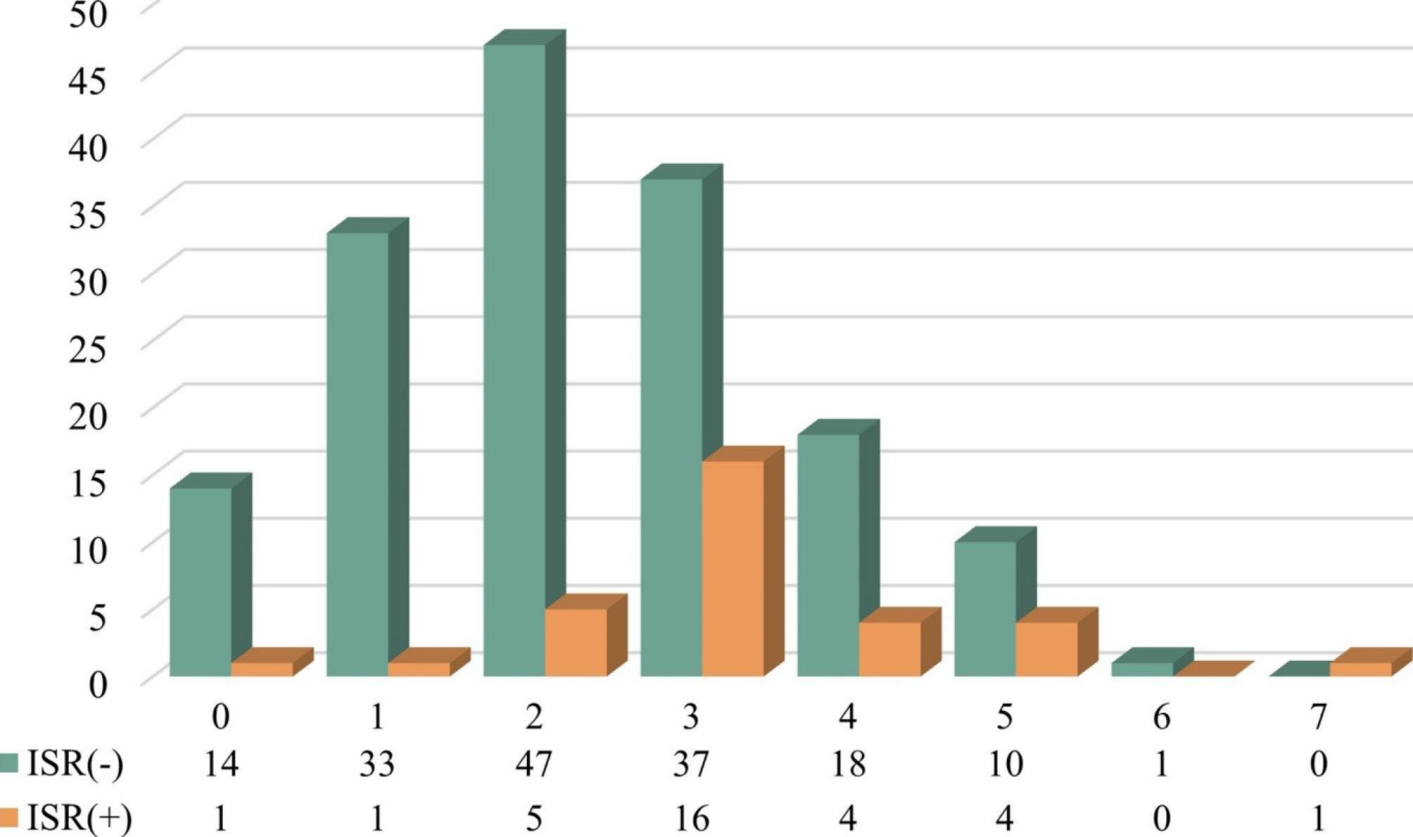
The distribution of new-generation drug-eluting stent (DES) in-stent restenosis (ISR) in patients according to different DES-ISR genetic risk scores

The distribution of DES-ISR GRS values ranged between 0 and 7 (Fig. 1). The majority of geriatric patients with DES-ISR had a GRS score ≥ 3 (Fig. 1). The optimal cut-off value of the DES-ISR GRS in geriatric East Asian patients was determined by performing a diagnostic accuracy test. After calculating the sensitivity, specificity, and accuracy, we defined the cut-off value of the DES-ISR GRS as ≥ 3 (Table 3).

**Table 3:** The overall predictive accuracy of the different cutoff values of DES-ISR GRS in the elderly

Cutoff value of DES ISR GRS	Sensitivity (%)	Specificity (%)	PPV (%)	NPV (%)	Accuracy (%)
≥ 2	93.75	29.38	20.98	95.92	40.10
≥ 3	78.12	58.75	27.47	93.07	61.98
≥ 4	28.12	81.88	23.68	85.06	72.92

Abbreviation: DES: drug−eluting stent; ISR: in−stent restenosis; GRS: genetic risk score; PPV: positive predictive value; NPV: negative predictive value

The cutoff value was used to divide the patients into two groups: low GRS (0–2) and high GRS (≥ 3). Univariate analysis revealed acute myocardial infarction (AMI) (p = 0.003) and high GRS (≥ 3) (p < 0.001) had significant correlations with the DES-ISR risk; multivariate analysis still showed that AMI (p = 0.008) and high GRS (p < 0.001) were associated with an increased risk of the DES-ISR (Table 4).

**Table 4:** The univariate and multivariate analysis to reveal the relation between covariate and the DES-ISR risk

	Univariate analysis		Multivariate analysis	
Variable	HR (95% CI)	*p*	HR (95% CI)	*p*
Age	0.981 (0.924–1.042)	0.54	0.979 (0.922–1.039)	0.48
Sex	1.072 (0.481–2.386)	0.87	1.125 (0.502–2.518)	0.78
AMI	2.869 (1.427–5.77)	0.003	2.573 (1.273–5.201)	0.008
GRS (≥ 3)	5.003 (2.163–11.57)	0.0001	4.569 (1.969–10.603)	0.0004

Abbreviation: AMI: acute myocardial infarction

**Fig. 2:**
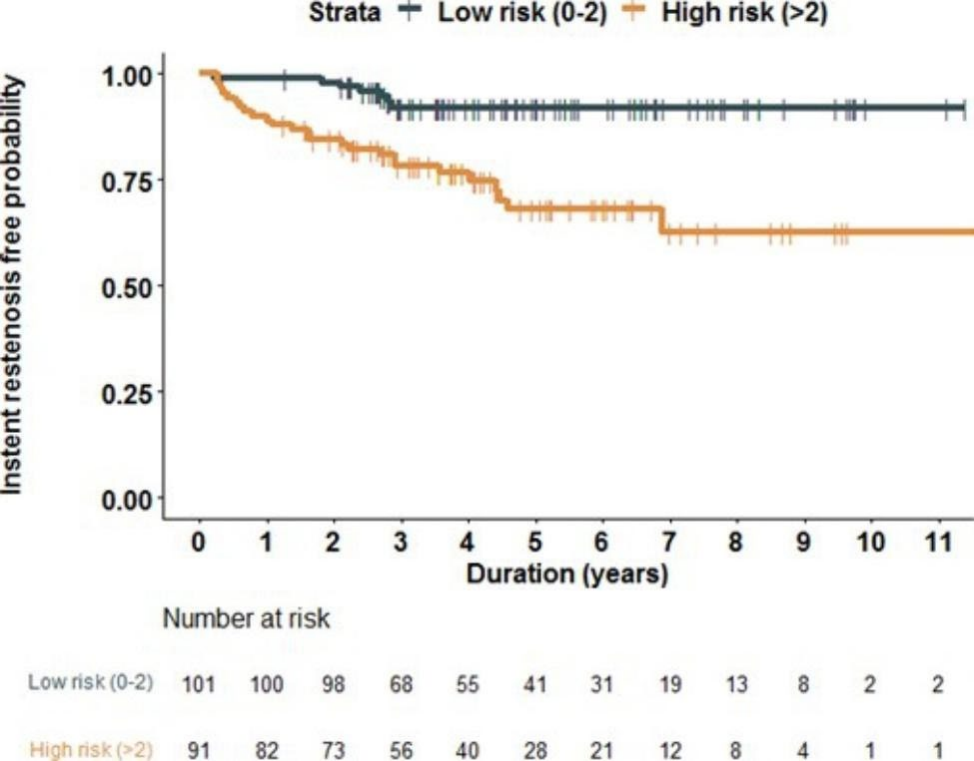
The Kaplan–Meier curve for drug-eluting stent (DES) in-stent restenosis (ISR) in geriatric patients with low (green) and high (orange) genetic risk scores (GRS)

Therefore, as mentioned earlier, we used propensity score matching to eliminate the effect of AMI. The Cox regression analysis revealed the risk of DES-ISR in patients with high GRS was significantly higher than that in patients with low GRS (HR 4.46, 95% confidence interval: 1.93–10.34, *p* = 0.0004). The DES ISR-free probability was estimated by the Kaplan-Meier method which was higher in the low GRS group (log-rank test *p* = 0.002, Fig. 2).

## Discussion

This study examined the power of the established five SNP-derived DES-ISR GRS model [3] in an older population. We demonstrated that this model was significantly associated with second-and third-generation DES-ISR in geriatric East Asian patients. Geriatric people with high GRS (≥ 3) are at increased risk of next-generation DES-ISR, demonstrating that in older people, genetic impact on the second-and third-generation DES-ISR still exists.

The main mechanism of DES-ISR is neointimal hyperplasia, which includes inflammation and vascular smooth muscle cell (VSMC) proliferation [1]. After coronary stent deployment, endothelial cell injury triggers a cascade of platelet, fibrin, and macrophage migration [1, 5, 6]. These processes aggregate blood cells, extracellular matrices (ECMs), and VSMCs, which produce chemokines, cytokines, and growth factors [1, 6]. Subsequently, an inflammatory environment is initiated, resulting in VSMCs migrating from the media to the intima and proliferating. Finally, reendothelialization develops to form neointima [6].

The proposed DES-ISR GRS model was shown to be significantly associated with second-and third-generation DES-ISR. These SNPs contribute to ISR as most of them are associated with VSMC proliferation. CAMLG is a signal transducer for angiotensin II in regulating the calcineurin-NFAT pathway and smooth muscle cell senescence [7]; GALNT2 activates the smooth muscle cell EGFR, leading to proliferation [8]; THOC5 participates in the M-CSF-induced macrophage differentiation, which causes VSMC de-differentiation and migration [9]; C11orf84 inhibits the expression of SNIP1 and the SNIP1-mediated Wnt signaling pathway [10], resulting in cell proliferation and migration; and SAMD11 plays a role in cell proliferation [11].

There are many factors that result in vascular aging, such as oxidative stress, mitochondrial dysfunction, vascular inflammation, maladaptation to molecular stress, loss of proteostasis, genomic instability, dysregulated nutrient pathways, and the renin-angiotensin system (RAS). These factors lead to vascular inflammation and contribute to functional dysregulation with pathological manifestations such as atherosclerosis, hypertension, and vascular impairment. Therefore, inflammation is fundamental to damage and degeneration of the older population’s vascular system [12].

Inflammation is essential for neointimal hyperplasia and VSMC proliferation. The blood vessels of older people are prone to chronic inflammation, resulting in neointimal hyperplasia. In the current study, we revealed the genetic impact of the five selected exonic SNPs on second-and third-generation DES-ISR in East Asian geriatric patients.

Disease prevention, treatment, and follow-up of older people are essential. Once DES-ISR occurs in older people, they are prone to fragility because of the associated comorbidities and the medical complications during revascularization. Several biological, mechanical, and technical factors have been proposed to illustrate DES ISR [5]. Although patients have the same disease, the severity, duration, and treatment effects of the disease may vary significantly. The lesion characteristics and stent designs are distinct. Although we tried our best to control all the mechanical and technical issues associated with deploying DES in diseased coronary arteries, DES-ISR still occurred. Consequently, we need to investigate genetic factors that could predict DES-ISR risk and provide more individualized treatment. Personalized medicine has emerged and is increasingly guiding treatment decisions. Therefore, in this study, we provided a genetic perspective on ISR risk in the older people population.

In many clinical situations, it is often difficult to determine whether elderly patients with CAD and multiple comorbidities should undergo percutaneous coronary intervention or coronary artery bypass grafting (CABG). Each procedure’s long-term benefit, complication risk, and patient preferences must be considered. The GRS model could provide a new perspective for discussing DES-ISR risks. This model would allow shared decision-making from a genetic perspective and personalized medicine according to the different “individualized risks” in addition to “group risks.”

Nonetheless, this study had some limitations. First, this was a retrospective cohort study from a single Taiwanese university medical center, with a small number of patients. The application and externalization of different ethnic or races are the unignorable issues of genetic studies. Every race has its own genetic risks to predict different disease outcomes. Therefore, our findings may not be applicable to other races. Thus, we recognized that it is necessary to test our DES-ISR GRS model in a larger population or with other races. Second, we did not conduct a validation cohort or an external validation analysis to confirm the validity of the GRS model in geriatric patients. Third, we did not perform a functional analysis to reveal the molecular mechanisms of the SNPs affecting aged blood vessels. Therefore, a larger prospective cohort involving multiple centers or different countries with validation cohorts is needed to further evaluate the GRS model in the geriatric population.

## Conclusion

We demonstrated that the GRS model, comprising five SNPs, is capable of predicting DES-ISR in geriatric patients with CAD. This model could provide genetic information for both patients and interventional cardiologists for deciding percutaneous coronary or surgical interventions.

## Data Availability

The datasets used and/or analyzed during the current study are available from the corresponding author on reasonable request.
